# A Case of Atypical Hemolytic Uremic Syndrome in a Pregnant Patient

**DOI:** 10.7759/cureus.25096

**Published:** 2022-05-18

**Authors:** Haider Ghazanfar, Iqra Nawaz, Nishant Allena, Shoaib Ashraf, Muhammad Saad, Nisha Ali

**Affiliations:** 1 Internal Medicine, BronxCare Health System, Bronx, USA; 2 Internal Medicine/Nephrology, Stony Brook University, Stony Brook, USA; 3 Internal Medicine, BronxCare Health System/Icahn School of Medicine at Mount Sinai, Bronx, USA; 4 Cardiology, BronxCare Health System/Icahn School of Medicine at Mount Sinai, Bronx, USA

**Keywords:** pregnancy, monoclonal antibodies, eculizumab, atypical hemolytic uremic syndrome, thrombotic microangiopathies

## Abstract

Atypical hemolytic uremic syndrome (HUS) is a rare but severe form of thrombotic microangiopathies (TMAs) that affects both children and adults. The clinical presentation is usually nonspecific, including a broad spectrum of symptoms ranging from abdominal pain, confusion, diarrhea, fatigue, irritability, hypertension, and lethargy. We present a case of a 36-year-old woman with medical comorbidities of asthma and pulmonary embolism who presented to our hospital in the 36th week of her pregnancy for preterm premature rupture of the membranes. The postoperative course was complicated with a sudden onset drop in hemoglobin and acute onset thrombocytopenia. Complements levels were normal while ADAMTS 13 (von Willebrand factor-cleaving protease) activity was 81% which ruled out ADAMTS 13 deficiency. No significant clinical improvement was seen after five cycles of plasmapheresis. She was later started on Eculizumab biweekly with marked improvement in biochemical and clinical status*. *Prompt diagnosis and treatment of atypical HUS are crucial as the prognosis is poor if untreated. The diagnosis of atypical HUS can be challenging as the classic triad of microangiopathic hemolytic anemia, thrombocytopenia, and acute kidney injury can be seen in all thrombotic microangiopathies, thus careful clinical and laboratory assessment is required to establish the diagnosis. The new treatment modality, Eculizumab, the anti-complement monoclonal antibody, has become the first-line therapy for treating atypical HUS.

## Introduction

Atypical hemolytic uremic syndrome is a rare but severe form of thrombotic microangiopathies (TMAs) that affects both children and adults. The reported incidence is between 0.42 and 1.9 per million population annually [1]. In the United States, the incidence of atypical hemolytic uremic syndrome (HUS) is approximately 1 in 500,000 people per year [2]. Studies conducted in Europe estimate the annual incidence between 0.23 and 1.9 per million annually [3]. The results of the systemic review have shown that the incidence of atypical HUS among individuals younger than 20 years old is approximately 0.26 to 0.75 per million population [1]. Atypical HUS accounts for only <10% of all cases of hemolytic uremic syndrome [1]. Females have a higher incidence than males since pregnancy is one of the primary triggers of atypical HUS. Atypical HUS presents during pregnancy, mainly in the postpartum period in genetically predisposed women [4]. It affects approximately 1 in 25,000 pregnancies and is usually associated with unfavorable outcomes [5].

Atypical HUS characteristically presents with thrombocytopenia and microangiopathic hemolytic anemia. The clinical presentation is usually nonspecific, including a broad spectrum of symptoms ranging from abdominal pain, confusion, diarrhea, fatigue, irritability, hypertension, and lethargy [6]. Patients often present with acute renal failure, with most patients (81% of adults and 59% of children) requiring hemodialysis [6]. Cardiovascular complications like myocardial infarctions and cardiomyopathies have also been reported in the literature [7]. Neurological complications include headaches, diplopia, transient ischemic attacks (TIAs), and seizures. Pulmonary symptoms include pulmonary edema and hemorrhage [7].

The prognosis of the atypical hemolytic syndrome is usually poor if untreated. Higher rates of kidney failure, progression into end-stage renal disease requiring hemodialysis, and other chronic serious complications such as severe high blood pressure are associated with atypical HUS compared to typical HUS [8].

## Case presentation

A 36-year-old woman with medical comorbidities of asthma and pulmonary embolism presented to our hospital in the 36th week of her pregnancy for preterm premature rupture of the membranes. Her past family history was unremarkable. She denied smoking, drinking alcohol, or using illicit substances. She was allergic to Bactrim (sulfamethoxazole and trimethoprim) and penicillin. At the presentation, she was in mild distress. Lung sounds were clear bilaterally. Abdominal examination was remarkable for Linea nigra and a slightly tender abdomen. Fetal sounds were present. She underwent emergent cesarean delivery due to breech presentation with hysterotomy and bilateral fimbriectomy. The postoperative course was complicated with a sudden onset drop in hemoglobin and acute onset thrombocytopenia requiring four units of packed red blood cells without significant improvement. The pelvis's computerized tomography (CT) without contrast showed a large complex pelvic hematoma measuring at least 18.5 x 8 x 12.8 cm with moderate right-sided hydronephrosis. The patient underwent an inferior vena cava filter, bilateral nephrostomy tube placement, and percutaneous abdominal wall collection sampling aspiration. Cultures from pelvic fluid drainage and nephrostomy sample showed no bacterial growth. On postoperative day 5, the patient was started on intravenous antibiotics after an episode of fever. The patient was transferred to the intensive care unit.

Heparin-induced thrombocytopenia was ruled out. The patient's peripheral blood smear showed schistocytes with elevated lactate dehydrogenase of 1659 units/L, low haptoglobin <10, anemia, low platelet count, and worsening renal function, (serum creatinine 3.4 mg/L) raising the suspicion of thrombotic microangiopathies. The patient's initial laboratory values are presented in Table 1.

**Table 1:** Laboratory values during the hospital course

Laboratory Parameter	Admission Day	Third Day of Admission	Fifth Day of Admission	Twelfth Day of Admission	Eighteenth Day of Admission	Reference Range
Hemoglobin (g/dl)	8.9	6.6	6.5	7.8	8.3	12-16
Hematocrit (%)	26.9	19.9	23	22.9	23.1	42-51
White Cell Count (per μl)	14.4	10.7	17.2	12.7	8.0	4.8-10.8
Neutrophils	87.2	87.4	83.1	77.4	70.2	40-70%
Lymphocytes	6.3	5.8	6.1	11.3	15.7	20-50%
Platelet Count (per μl)	176	97	70	82	75	150-400
Urea nitrogen (mg/dl)	7	19	20	51	74	6-20
Creatinine (mg/l)	0.8	1.2	1.4	3.4	6.4	0.5-1.5
Lactate dehydrogenase (unit/L)		702	991	2856	934	100-190

The patient was transfused four units of packed red blood cells, four units of fresh frozen plasma, two packs of platelets, and one unit of cryoprecipitate, intravenous fluids, with no significant blood count improvement. Renal ultrasound was obtained which did not show any evidence of hydronephrosis or obstruction. The patient remained non-oliguric throughout the hospital course. Complement studies revealed a C3 level of 147 (normal range: 90 to 150 mg/dL) and a C4 level of 30 (normal range: 16 to 47 mg/dL). ADAMTS 13 (von Willebrand factor-cleaving protease) was positive at 81%. The patient had a negative direct and indirect Coombs test. Renal biopsy was not done. After five sessions of plasmapheresis, no significant clinical improvement was seen and she was transferred to the tertiary care center. She was later started on Eculizumab biweekly with marked improvement in biochemical and clinical status. Based on these findings, classical atypical HUS was diagnosed. The patient's clinical condition improved, and the laboratory values stabilized. The patient was given follow-up appointments.

## Discussion

**Figure 1:**
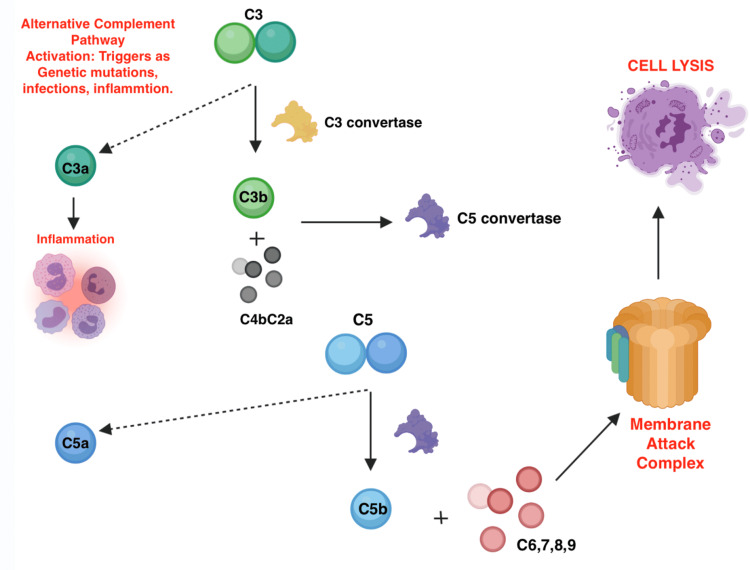
Illustration of mechanism of alternative complement pathway

Pregnancy-associated aHUS is a severe systemic disease associated with high morbidity and mortality. The diagnosis of pregnancy-associated aHUS remains very challenging as the pathogenesis and presentation is not very well understood and still remains ill-defined in literature. The proposed mechanism is the overactivation of the complement pathway, which results in endothelial damage leading to the formation of the platelets and fibrin microthrombi in the vessels, which subsequently leads to hemolytic anemia, thrombocytopenia, and ischemia, causing multiorgan damage, including acute renal injury [9]. The steps involved in the activation of inflammatory cascade via alternative complement pathway are further depicted in Figure 1 below.

Atypical HUS is thought to be manifested as a genetic predisposition caused by the combined effect of various gene mutations and environmental factors. During pregnancy, multiple risk factors such as inflammation, drugs, preeclampsia, maternal-fetal hemorrhage, infections, placental abruption, spontaneous abortion, etc., can result in the overactivation of the alternative complement pathway [10]. The most commonly reported genetic mutation is the CFH gene mutation, which encodes the formation of factor H. Factor H protects blood vessels from injury and endothelial damage and thus prevents the overactivation of an inflammatory cascade [2]. Other genes involved are endothelial anticoagulant glycoprotein thrombomodulin (THBD) gene, loss of function mutations of factor I, membrane cofactor protein (MCP), and gain of function mutations of factor B and factor C3 [2].

Differentiating atypical HUS from other thrombotic microangiopathies is imperative for establishing a prompt diagnosis, appropriate management and to prevent morbidity and mortality. However, differentiating pregnancy-associated atypical HUS from other pregnancy-related complications such as HELLP syndrome, preeclampsia, and acute fatty liver of pregnancy can be challenging [10]. Hemolytic anemia, acute renal failure, and thrombocytopenia are the standard features that can be seen in any of these syndromes, making it diagnostically difficult to differentiate from each other [10]. Also, triggers such as HELLP syndrome and preeclampsia can easily predispose pregnant patients to rapidly progress to atypical HUS and thrombotic thrombocytopenic purpura (TTP). Mutations in complement regulatory proteins have also been reported in HELLP syndrome, causing a diagnostic dilemma [11]. The comparison of various factors which can help differentiate A-HUS and TTP from HELLP syndrome and acute fatty liver of pregnancy is presented in Table 2 below.

**Table 2:** Comparison of various diagnostic factors among A-HUS, TTP, AFLP and HELLP/Preeclampsia HUS: Hemolytic uremic syndrome; TTP: Thrombotic thrombocytopenic purpura; HELLP: Hemolysis, elevated liver enzymes, low platelet count; AFLP: Acute fatty liver of pregnancy; LDH: Lactate dehydrogenase; INR: International normalized ratio; PTT: Partial thromboplastin time; AKI: Acute kidney injury.

	Atypical HUS	TTP	HELLP/Preeclampsia	Acute fatty liver of pregnancy (AFLP)
Time of presentation	Postpartum	2nd and 3rd trimester	3rd trimester	3rd trimester
Clinical features	Abdominal pain, nausea/vomiting hypertension +/-	Purpura fever, altered mental status/seizures	Hypertension, abdominal pain, nausea/vomiting jaundice +/-	Jaundice, abdominal pain, nausea/vomiting, malaise
Significant lab findings	Hemolytic anemia (high LDH, low haptoglobin), Thrombocytopenia -severe AKI ->10% ADAMTS13 activity	Hemolytic anemia (high LDH, low haptoglobin) Thrombocytopenia -<10% ADAMTS13 activity - Mild AKI	Mild anemia (Hemolytic Anemia (high LDH, low haptoglobin) Thrombocytopenia -Proteinuria -Transaminitis -Hyperbilirubinemia - Mild AKI	Anemia thrombocytopenia - Elevated PTT -coagulopathy (elevated INR) --Hypoglycemia -Elevated ammonia -Transaminitis -Hyperbilirubinemia - Moderate AKI
Recovery	No recovery after delivery	No recovery after delivery	1 week after delivery	1-2 days after delivery

Multiple laboratory entities can help to establish the most likely diagnosis. For example, the key differentiator of atypical HUS from thrombotic thrombocytopenic purpura (TTP) is the ADAMTS 13 activity level. ADAMTS 13 activity level is mostly <5% in TTP and >5 - 10% in atypical HUS. Other laboratory investigations such as complement regulatory protein levels like CH50, FH, and FI, anti-FH antibody levels are also commonly used [4]. C5a and C5b-9 serum levels are typically elevated in atypical HUS [12]. A Shiga toxin PCR or ELISA is also obtained to rule out typical HUS. According to a study, ADAMTS 13 level is a valuable diagnostic tool and a good marker of prognosis in patients undergoing plasma exchange therapy. It showed that elevated ADAMTS 13 levels are associated with better outcomes and can recognize poor results and guide advancement to other treatment modalities [12]. The comparison of various factors which can differentiate among Typical HUS, atypical HUS, and TTP are presented in Table 3 below.

**Table 3:** Comparison of various diagnostic factors among T-HUS, A-HUS and TTP VWF: von-Willebrand factor; STEC: Shiga toxin secreting strain of E. coli; ESRD: End-Stage Renal Disease

	Typical Hemolytic Uremic Syndrome (T-HUS)	Atypical Hemolytic Uremic Syndrome (A-HUS)	Thrombotic Thrombocytopenia Purpura (TTP)
Mode	Acquired	Hereditary Idiopathic	Hereditary/Acquired – Autoimmune
Etiology	Shiga/verotoxin mediated: Enteric infection via Shiga toxin secreting strain of E. coli (STEC), Shigella dysenteriae	Mutations in genes encoding proteins of alternative complement pathway	Genetic mutations causing ADAMTS13 deficiency or autoantibodies inhibition of ADAMTS13
Pathophysiology	Toxin-mediated endothelial damage	Defective complement regulation	Endothelial damage and thrombosis due to ultra large VWF multimers
Affected age group	Children <5 yo	Children/Adults	Inherited: Neonatal Acquired: Adults
Clinical Presentation	Gastrointestinal symptoms: nausea, vomiting, abdominal pain, diarrhea, acute kidney injury, hemolytic anemia, thrombocytopenia, organ failure	Acute kidney injury, hemolytic anemia, thrombocytopenia, organ failure	Neurological symptoms: confusion, seizures. Acute kidney injury, hemolytic anemia, thrombocytopenia, organ failure
Histologic Features	Schistocytes, microvascular thrombosis, microangiopathy	Schistocytes, microvascular thrombosis, microangiopathy	Schistocytes, microvascular thrombosis, microangiopathy
Diagnostic test	Shiga toxin/STEC +ive	ADAMTS13 > 5-10	ADAMTS13 <5%
Treatment	Supportive care	Plasma infusion/exchange or Anticomplement therapy (Eculizumab)	Plasma infusion/exchange, immunosuppressive therapy (rituximab, cyclophosphamide), splenectomy
Prognosis	>12% ESRD or death if untreated	>50% ESRD or death if untreated	>90% death if untreated

Treatment modalities in atypical HUS are mainly directed towards deteriorating renal function. Current treatment options include plasmapheresis, the novel anti-complement therapy, dialysis, and renal transplantation [13]. Current guidelines by the American Society of Apheresis outline that it is reasonable to initiate plasma exchange given high suspicion for atypical HUS while awaiting the ADAMTS 13 results. Improved survival rates have been shown in patients treated with plasma exchange (PEX) [13]. The efficacy of plasma exchange is notably seen if the CHF gene or MCP gene mutation is the cause of atypical HUS - as plasma exchange can help remove the antibodies to CHF/MCP gene [14].

In cases of atypical HUS, anti-complement therapy is appropriate, and Eculizumab is currently recommended for the treatment. It is a high affinity humanized recombinant monoclonal antibody that prevents the activation of terminal complement cascade by binding to complement protein C5 and blocking its cleavage, thereby inhibiting its pro-inflammatory, prothrombotic lytic products, which include c5a and membrane attack complex, which is c5b-9 [15]. Its effectiveness has been evidenced by normalizing haptoglobin levels, increasing platelet counts, and decreasing creatinine levels [16]. Its use has also been proven to be safe in pregnancy with no reported fetal complications [17]. Dialysis is initiated when deterioration in renal function meets the indications for dialysis, and renal transplantation is also a modality of treatment for atypical HUS but has been complicated by recurrence and resulting in graft loss from thrombotic microangiopathy. The use of anti-complement (C5) antibody therapy such as Eculizumab might have a possible role in preventing such outcomes [18]. Patients treated with Eculizumab have shown improved renal function based on estimated glomerular filtration rate (eGFR) and remained stable for up to six years. In those who discontinued the treatment, a decrease in renal function was noted [15]. Among all other benefits listed, Eculizumab has also been proven to improve the overall quality of life.

## Conclusions

The atypical HUS is a rare but one of the most severe forms of thrombotic microangiopathies (TMAs). The ADAMTS-13 activity level plays a crucial role in diagnosing, treating, and prognoses of atypical HUS. Plasma exchange is the first line of treatment and can be initiated while awaiting the ADAMTS-13 activity level. As per the American Society of Hematology guidelines if ADAMTS-13 activity levels are >10% and if there is no notable hematological improvement after five days, it is reasonable to discontinue plasma exchange therapy and recommend to start anticomplement therapy - Eculizumab. Prompt diagnosis and treatment of atypical HUS are crucial as the prognosis is poor if untreated. The diagnosis of atypical HUS can be challenging as the classic triad of microangiopathic hemolytic anemia, thrombocytopenia, and acute kidney injury can be seen in all TMAs. Careful clinical and laboratory assessment is required to establish the diagnosis.
